# Comparable detection of nasopharyngeal swabs and induced sputum specimens for viral nucleic acid detection of suspected novel coronavirus (SARS-Cov-2) patients in Fayoum governorate, Egypt

**DOI:** 10.1186/s43088-023-00379-4

**Published:** 2023-05-02

**Authors:** Doaa Y. Ali, Rasha A. Hussein, Shahira Morsy ELshafie, Reem Amgad Mohamed, Fadwa Abd El Reheem

**Affiliations:** 1 Department of Clinical and Chemical Pathology, Faculty of Medicine, Fayoum University, Fayoum, Egypt; 2 Department of Medical Microbiology and Immunology, Faculty of Medicine, Zagazig University, Zagazig, Egypt

**Keywords:** Induced sputum, Nasopharyngeal swab, SARS-CoV-2, RT-qPCR, Viral load

## Abstract

**Background:**

The most commonly utilized samples for severe acute respiratory syndrome coronavirus 2 (SARS-CoV-2) detection using real-time quantitative reverse transcriptase-polymerase chain reaction (RT-qPCR) are nasopharyngeal swabs (NPS) and oropharyngeal swabs. However, there are some drawbacks. For SARS-CoV-2 detection, induced sputum might be analyzed and may be equivalent to pharyngeal swabs. This study was done to assess the potential superiority of induced sputum over NPS for SARS-CoV-2 detection. Sixty symptomatic COVID-19 patients who attended Fayoum University Hospitals in Fayoum Governorate, Egypt, were included in this cross-sectional descriptive study. Paired NPS and induced sputum samples were collected from each subject on the third and tenth days after symptoms began for RT-qPCR SARS-COV2 diagnosis.

**Results:**

At day 3, 52 (86.7%) of NPS and 48 (80.00%) of induced sputum specimens had positive RT-qPCR results with a significant statistical difference (*P* = 0.001). At day 10, 41 induced sputum samples (68.3%) were negative, while 19 (31.7%) were positive. Only three (5.0%) of the 19 positive induced sputum samples tested positive for NPS. NPS samples had a higher viral load than induced sputum samples at day 3 [25 (41.7%) vs. 23 (38.3%)]. At day 10, induced sputum samples had a higher viral load than NPS [9 (15.0%) vs. 6 (10.0%)]. A statistically significant positive correlation between the viral load value of the NPS and the induced sputum sample at day 3 (r = 0.497, *p* = 0.00) denoting similarity in the results of the two types of samples. By ROC analysis, the highest area under the curve for the overall CT value of the induced sputum was (0.604), with a statistically significant difference (*p* value = 0.0418).

**Conclusion:**

In the early stages of the disease, induced sputum and NPS tests had comparable results, but NPS yielded more false negative results later in the disease course than an induced sputum sample, which yielded higher sample positivity and viral load than NPS. Furthermore, induced sputum collection is a straightforward, non-invasive, and risk-free method. As a result, induced sputum could be useful for COVID-19 confirmation in patients with radiologically or epidemiologically suspected COVID-19 who have a negative NPS or in difficult-to-diagnose COVID-19 patients.

## Background

The World Health Organization (WHO) has identified the '2019 new coronavirus,' or 'COVID-19,' as the primary cause of the current pneumonia epidemic, which began in early December 2019 in Wuhan, Hubei Province, China [1]. On February 14, 2020, Egypt reported its first case of coronavirus disease 2019 (COVID-19) [2].

Coronaviruses belong to the Nidovirales order, the Coronaviridae family, and the Coronavirinae subfamily of the Nidovirales order. Based on serological data, the Coronavirinae are divided into four families: alphacronavirus, betacoronavirus, gammacoronavirus, and delta coronavirus [3]. Severe acute respiratory syndrome coronavirus 2 (SARS-CoV-2), the agent responsible for COVID-19, is a species of the genus Betacoronavirus [3].

The gold standard approach for detecting SARS-CoV-2 has always been probe-based Real-time quantitative reverse transcriptase-polymerase chain reaction (RT-qPCR), which the Centers for Disease Control and Prevention (CDC) and WHO recommend for population screening globally [4].

The RNA-dependent RNA polymerase (RdRp) gene, envelope (E) gene, spike (S) gene, nucleocapsid (N) gene, and ORF8 or ORF1b portions of the SARS-CoV-2 genome were all targeted using RT-qPCR assays [5].

For a Covid-19 diagnosis, the upper respiratory tract is sampled from the nasopharyngeal (NPS), oropharyngeal swab (OPS), NP wash, or saliva, while the lower respiratory tract is sampled from sputum, tracheal aspirate, bronchoalveolar lavage fluid (BAL), or bronchoscopic brushing [6, 7]. Which are considered to be even more sensitive than upper respiratory tract samples [6].

The most commonly utilized samples for COVID-19 detection using RT-qPCR are NPS and OPS. However, there are some drawbacks, such as the false-negative findings in upper respiratory samples obtained from asymptomatic patients or mild illnesses, as well as the necessity for repeat sampling and testing [7]. Negative findings do not rule out the possibility of COVID-19 infection [8]. Nasopharyngeal RT-PCR positivity is thought to decline within one week of symptom onset; therefore, a positive test late in the course of the disease is expected to be from sputum, BAL [9]. So, the need for appropriate specimen selection is critical for increasing SARS-Cov-2 detection using the RT-qPCR technique and lowering existing false-negative detection. As a result, searching for alternative specimen types with greater precision and detection accuracy is required.

Sputum is a helpful, non-invasive technique for the detection of SARS-CoV-2. It might identify SARS-CoV-2 at a greater rate than NPS or throat swabs [10, 11]. A possible drawback of testing sputum samples for COVID-19 diagnosis is that not all patients infected with COVID-19 can expectorate sputum, and it is restricted to patients who can introduce sputum. According to certain research, low sputum production is a prevalent symptom in COVID-19 patients, making it difficult to get sputum in these individuals [12, 13]. So, for SARS-CoV-2 detection, induced sputum or a self-collected deep cough specimen might be analyzed, especially in those who are uncapable of introducing sputum, and may be equivalent to pharyngeal swabs [14, 15]. Sputum induction using a hypertonic saline solution is one of the most commonly used methods for studying airway secretions in patients with lung disorders [16], such as chronic obstructive pulmonary disease, bronchial asthma, and pulmonary hypertension [17]. Recently, in Mycoplasma pneumoniae and infantile tuberculosis [18].

The safety and effect of induced sputum for SARS-CoV-2 detection had previously been reported in some multi-center cross-sectional studies [19, 20]. Therefore, we conducted this study to further assess the potential superiority of induced sputum over NPS for SARS-CoV-2 detection.

## Method

### Study design

This is a cross-sectional descriptive study that was conducted on sixty symptomatic COVID-19 patients who attended Fayoum University Hospitals, Fayoum Governorate, Egypt, from April 30 to October 30, 2021.

The study follows the Declaration of Helsinki guidelines. In addition, informed written consent was obtained from the legal guardians of all subjects participating in this study. The severity classification of our cases was according to WHO interim guidance into mild, moderate, severe, and critical cases [21].

We included in our study all suspected COVID-19 patients of both genders. While, we excluded asymptomatic patients, those diagnosed with SARS-CoV-2 infection via a single type of specimen (induced sputum or NPS), and any ventilated patients who could not give induced sputum samples.

All patients were subjected to a full medical history and clinical examination including the presence of any respiratory distress signs. On the third day after the onset of symptoms, all patients had a computed tomography of the chest (CT-Chest). Depending on the CT result, the patients were classified according to the coronavirus disease 2019 (COVID-19) Reporting and Data System (CO-RADS) classification from CO-RAD1 to CO-RAD6 and according to the total severity score (TSS) into minimal, mild, moderate, and severe degrees [22].

### Laboratory investigations

The following laboratory investigations were done on all patients on the first day of the onset of the symptom: Full blood examination including: total leucocytic count (TLC), platelets count, and hemoglobin (HB) (All were done on Sysmex XN 1000, Canada), C‐reactive protein (CRP) was done by automated CRP instrument (CoaDATA 4004 instrument,Germany), D-dimer was measured using a chemiluminescent enzyme immunoassay quantitative technique by (Path fast compact immune-analyzer, Japan).

### Samples

Each subject was asked to give paired samples of NPS and induced sputum at the 3rd and 10th days of the beginning of symptoms for SARS- COV-2 laboratory diagnosis by RT-q PCR. NPS were collected using specialized dacron, rayon, or calcium alginate-tipped collection swabs with plastic or non-aluminum wire shafts. Samples were collected in specific vials containing viral transport media (VTM) (STOR-F (DNA technology, Russia).

Each patient inhaled a 3% saline solution by nebulizer to induce sputum. In order to decrease the risk of oral contamination before induction of sputum, a saline solution was used to rinse the mouth, and then the patients were asked to give a deep cough and introduce sputum into a screw-capped sterile container. We followed the CDC's guidelines for collecting, handling, and testing COVID-19 different clinical specimens [23].

### RT-qPCR detection of SARS-COV-2

The molecular diagnosis of COVID-19 was done in the molecular biology unit in the Clinical and Chemical Pathology Department at Fayoum University Hospitals. Covid-19 RNA extraction was done by a nucleic acid extraction kit ("DNA- Technology ' made PREP-NA DNA/RNA) using Lab Turbo 48 C automated extraction system (Tiagen Bioscience Corporation, Taiwan). The SARS-CoV-2/SARS-CoV Multiplex REAL-TIME PCR Detection Kit is used to amplify and detect the SARS-COV-2 target region (N gene and ORF gene).

The following were RT-qPCR thermocycling conditions: for twenty minutes at 35 °C and for five minutes at 95 °C, fifty amplification cycles at 94 °C for ten seconds and 64 °C for fifteen seconds, then for one minute at 80 °C using DT Lite thermocycler (DNA Technology Research & Production, LLC, Russia). The quantification of viral nucleic acid in patient samples was done by measuring the RT-PCR cycle threshold (CT) of the ORF gene. The CT represents the number of replication cycles required to produce a fluorescent signal. The viral load is reflected by the CT number. CT value less than 25 indicates high viral load; CT value 25 to 35 indicates moderate viral load; and CT value greater than 35 indicates low viral load. To ensure validation of the result, an internal control is measured parallel to each sample, and positive and negative controls were used in each PCR run.

### Statistical analysis

Our study data was statistical analysis using SPSS (Social Science version 28.00). Descriptive analyses as frequency and percent for qualitative data and for quantitative data as median and interquartile range (IQR). Non-parametric quantitative data analysis was done using the Kruskal–Wallis test and the Mann–Whitney test. A qualitative analysis of associations between variables was compared with the Chi-square test. The receiver operating characteristic (ROC) and Spearman correlation coefficient (r) between the two variables were done. Statistical significance level at a *p* value ≤ 0.05.

## Results

### Demographic, clinical, and radiological data of the studied patients

This descriptive cross-sectional study included 60 clinically and laboratory-suspected COVID-19 patients, whose ages ranged from 15 to 72 years with a median age of 32 years. Twenty-one patients (35.0%) were male. Thirty-nine patients (65.0%) were female. Regarding COVID-19 risk factors, there were 7 patients (11.7%) with both diabetic mellitus (D.M.) and hypertension (HTN), while only 2 patients (3.3%) presented with chronic liver disease. The median of O2 saturation was 96.0%; it ranged from 80.0% to 99.0%. Thirty six patients (60.0%) had an abnormal CT finding. According to TSS, there were 7 cases (11.7%) in each mild and sever degree, and 10 cases (16.7%) had a moderate degree. The clinical data of our cases were detailed in (Table 1).

**Table 1:** Demographic, clinical, and radiological data of the studied patients

Age	Range	15.00–72.00	
	Median *(IQR)*	32.00 (25.00 45.00)	
		Number	Percent (%)
Sex	Male	21	35.0
	Female	39	65.0
HCW	Non HCW	30	50.0
	HCW	30	50.0
HTN	Non-HTN	53	88.3
	HTN	7	11.7
DM	Non DM	53	88.3
	DM	7	11.7
CLD	Non-hepatic patients	58	96.7
	Hepatic patients	2	3.3

Clinical symptoms		Number	Percent (%)
Fever	Absent	11	18.3
	Present	49	81.7
Cough	Absent	13	21.7
	Present	47	78.3
Dyspnea	Absent	45	75.0
	Present	15	25.0
Anosmia	Absent	46	76.7
	Present	14	23.3
Diarrhea	Absent	54	90.0
	Present	6	10.0
CT. Finding classification according to TSS	No CT. finding	36	60.0
	Mild	7	11.7
	Moderate	10	16.7
	Severe	7	11.7
O2 saturation%	Range	80.0–99.0%	
	Median *(IQR)*	96.00% (94.00–97.00)	

Total Number = 60

*HCW* healthcare workers, *DM* diabetes mellitus, *HTN* hypertension, *CLD* chronic liver disease, *C.T* computed tomography, *TSS* total severity score, *IQR *Inter quartile range.

According to the COVID-19 severity classification, we classified patients into four groups: 35 cases (58.3%) were mild, 16 cases (26.7%) were moderate, and 9 cases (15.0%) were severe. No critical cases were presented in our study.

Regarding the laboratory results in our study, the medians of TLC, lymphocytic count, HB, platelet count, CRP, and D-dimer were (6.22, 2.10, 12.55, 234.50, 5.50, and 0.30) respectively (Table 2).

**Table 2:** Laboratory investigations of the studied patients

TLC (× 10^9^ per L)	
Range	2–25.9
Median (*IQR*)	6.22 (4.72–7.77)
Lymphocyte count (× 10^9^ per L)	
Range	0.55–4.50
Median (*IQR*)	2.10 (1.29–2.50)
Hb (g∕dL)	
Range	9.00–16.30
Median (*IQR*)	12.55 (11.50–13.57)
Platelet (× 10^9^ per L)	
Range	83.00–610.00
Median (*IQR*)	234.500 (200.00–292.50)
CRP (mg/L)	
Range	0.500–134.90
Median (*IQR*)	5.50 (3.00–36.22)
D-dimer (µg/mL)	
Range	0.10–5.00
Median (*IQR*)	0.30 (0.20–0.57)

*TLC* total leucocytic count, *H.B* hemoglobin, *CRP* C-reactive protein, *IQR* interquartile range

At day 3 of the beginning of the symptoms, the majority of the patients had positive RT-qPCR results, with 52 (86.7%) of their NPS and 48 (80.0%) of their induced sputum specimens being positive (Table 3). However, six patients (10.0%) showed positive NPS and negative induced sputum samples, while only two patients (3.3%) showed negative NPS and positive induced sputum samples (Table 4). The findings showed that positive rates displayed a highly significant statistical difference (*P* = 0.001) between NPS and induced sputum specimens at day 3.

**Table 3:** Distribution of RT-qPCR results on NPS and induced sputum samples

RT-qPCR results	Number	Percent (%)
NPS at day 3		
Negative	8	13.3
Positive	52	86.7
Induced sputum samples at day 3		
Negative	12	20.0
Positive	48	80.0
NPS at day 10		
Negative	46	76.7
Positive	14	23.3
Induced sputum samples at day 10		
Negative	41	68.3
Positive	19	31.7

Total N = 60

*RT-qPCR* real-time quantitative reverse transcriptase-polymerase chain reaction, *NPS* nasopharyngeal swabs

**Table 4:** Comparison of RT-qPCR results between NPS and induced sputum samples at day 3 and day 10

	Induced Sputum at day 3				Chi-square	*P*-value
	Negative		Positive			
	N	%	N	%		
*NPS at day 3*						
Negative	6	10.0	2	3.3	17.452	0.001 HS
Positive	6	10.0	46	76.7		

	Induced Sputum at day 10				Chi-square	*P*-value
	Negative		Positive			
	N	%	N	%		
*NPS at day 10*						
Negative	30	50	16	26.7	0.885	0.347 NS
Positive	11	18.3	3	5.0		

*NPS* nasopharyngeal swabs, *P*-value < 0.05 was considered statistically significant

*NS* non-significant at *p*-value > 0.05, *HS* high-significant

While, at day 10, 41 induced sputum samples (68.3%) were negative, while 19 (31.7%) were positive. Fourteen cases (23.3%) were positive by NPS (Table 3). From 19 positive induced sputum samples, only 3 cases (5.0%) were positive by NPS (Table 4). There was a statistically non-significant difference (*P* = 0.347) between the RT-qPCR results of NPS and induced sputum specimens at day 10.

**Fig. 1:**
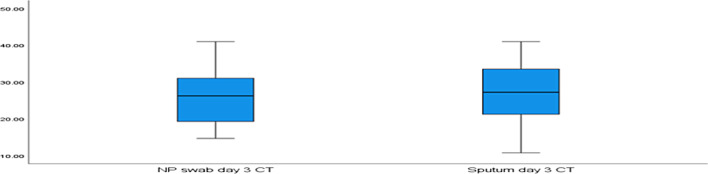
A boxplot comparing NPS and induced sputum samples at day 3 based on the cycle threshold (CT) value. Legend: Data are expressed as a box plot. The median (Inter quartile range) for the cycle threshold (CT) value of NPS at day 3 was (26.25) and for induced sputum samples was (27.25)

**Fig. 2:**
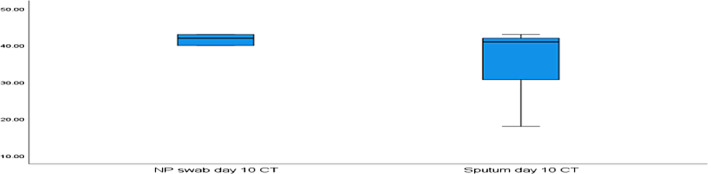
A boxplot comparing NPS and induced sputum samples at day 10 based on the cycle threshold (CT) value. Legend: Data are expressed as a box plot. The median (Inter quartile range) for the cycle threshold (CT) value of NPS at day 10 was (42.00) and for induced sputum samples was (41.00)

The median (IQR) for the NPS CT value at day 3 and at day 10 were (26.25 and 42.00), respectively, and for induced sputum samples at day 3 and day 10 were (27.25 and 41.00), respectively. The cycle threshold (CT) values of the NPs and induced sputum samples were non-significantly different at days 3 and 10 (*p* = 0.308 and 0.551, respectively) (Figs. 1, 2).

Regarding the viral load results, NPS samples had a higher viral load than induced sputum samples at day 3 [25 (41.7%) vs. 23 (38.3%)]. In contrast, at day 10, the results of the high viral load of induced sputum samples were superior to those of NPS [9 (15.0%) vs. 6 (10.0%)] (Table 5).

**Table 5:** Viral load classification of NPS and induced sputum samples at day3 and day10

	Number	Percent (%)
Viral load of NPS at day 3		
High viremia	25	41.7
Moderate viremia	27	45.0
Low viremia	8	13.3
Viral load of induced sputum samples at day3		
High viremia	23	38.3
Moderate viremia	24	40.0
Low viremia	13	21.7
Viral load of NPS at day 10		
High viremia	6	10.0
Moderate viremia	8	13.3
Low viremia	46	76.7
Viral load of induced sputum samples at day10		
High viremia	9	15.0
Moderate viremia	10	16.7
Low viremia	41	68.3

*NPS* nasopharyngeal swabs

**Fig. 3:**
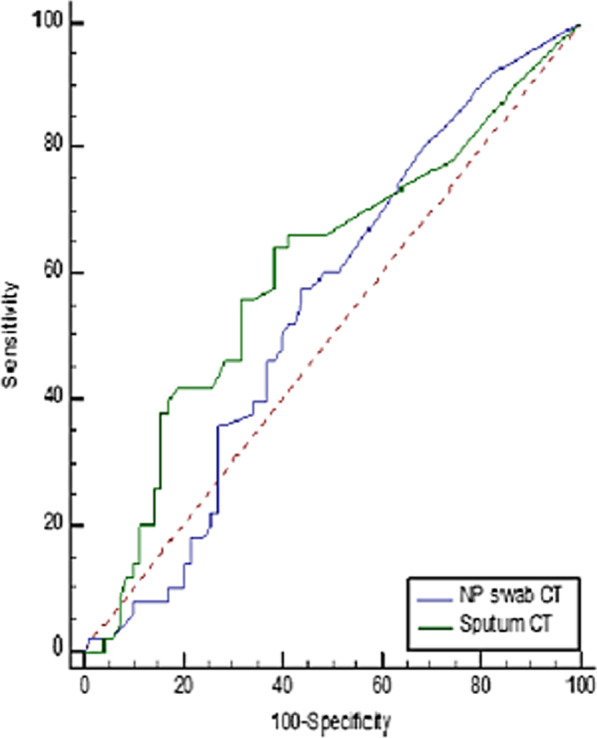
Receiver operating characteristic (ROC) curve for overall cycle threshold (CT) value of NPS and induced sputum samples. Legend: By ROC analysis, the area under the curve (AUC) for the overall CT value of the induced sputum samples was 0.604, with the best cutoff value at ≤ 32, sensitivity = 64.0% (95% CI, 49.2–77.1), specificity = 61.43% (95% CI, 49.0–72.8), and a *p* value = 0.0418 . The AUC for the overall CT value of NPS was 0.548, with the best cutoff value at ≤ 31.9, sensitivity = 58.0% (95% CI, 43.2–71.8), specificity = 55.71% (95% CI, 43.3–67.6), and *p* value = 0.3651

The receiver operating characteristic (ROC) curve was drawn to compare the overall diagnostic performance of NPS and induced sputum specimens during the study. By ROC analysis, we found that the highest area under the curve (AUC) for the overall CT value of the induced sputum samples was (0.604), which was greater than that of NPS (0.548) (Fig. 3), with a statistically significant difference (*p* value = 0.0418). The sensitivity of induced sputum samples was 64.0% (95% CI, 49.2–77.1), the specificity was 61.43% (95% CI, 49.0–72.8), the positive predictive value (PPV) was 54.2%, and the negative predictive value (NPV) was 70.5%. (Table 6; Fig. 3).

**Table 6:** Overall diagnostic performance of NPS and induced sputum samples

Cutoff point	AUC (95% Cl)	*P*-value	Sensitivity (95% Cl)	Specificity (95% Cl)	PPV (%)	NPV (%)
Overall CT of NPS						
≤ 31.9	0.548 (0.444–0.651)	0.3651	58.0% (43.2–71.8)	55.71% (43.3–67.6)	48.3	65.0
Overall CT of induced sputum samples						
≤ 32	0.604 (0.499–0.709)	0.0418	64.0% (49.2–77.1)	61.43% (49.0–72.8)	54.2	70.5

*AUC* area under the curve, *CT* cycle threshold, *CI* confidence interval, *PPV* Positive predictive value, *NPV* negative predictive value

**Fig. 4:**
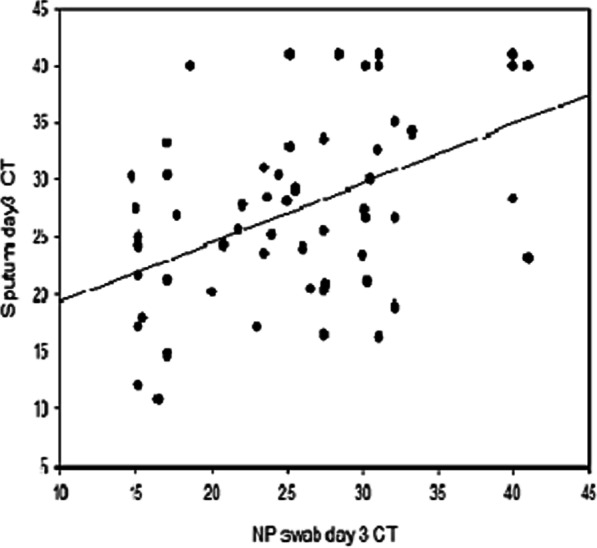
Linear correlation between the cycle threshold (CT) value of NPS and induced sputum samples at day 3. Legend: A correlation analysis was done between CT values of NPS and induced sputum samples at day 3, with a significant positive correlation (r = 0.497, *p* = 0.000)

By applying Spearman’s correlation test, there was a statistically significant positive correlation between viral load according to the CT value of the NPS and the induced sputum samples at day 3 (r = 0.497, *p* = 0.000), denoting similarity in the results of the two types of samples (Fig. 4).

## Discussion

The real-time reverse transcription-polymerase chain reaction assay (RT-q PCR) is the most important method for laboratory confirmation of SARS-CoV2. Nasopharyngeal swab remains the most common samples for SARS-COV-2 diagnosis but it has some limitations and easy to miss the diagnosis [7, 8]. Currently, there are more instances of false negative nucleic acid testing findings and released patients who turn positive again are increasing [24]. As a result, it may be necessary to reconsider whether the patients are virally free. SARS-CoV-2 detection through RT-qPCR might be enhanced by improving specimen selection, which is important to minimize the number of false negative results.

Therefore, looking for other specimen types with greater accuracy and detection efficiency is necessary. So, we conducted this research to assay the potential superiority and the accuracy of induced sputum over NPS for viral nucleic acid detection of novel coronavirus (SARS-Cov-2). This study was done at Fayoum University Hospitals and included 60 patients who fulfilled the inclusion criteria and formed the study population. We measured the viral load by nucleic acid assays (RT-qPCR) in paired samples (Nasopharyngeal swabs and induced sputum samples) early on day 3 to ensure having samples with a high viral load, and on day 10 to confirm that patients are free from infection and can return to their work; this is according to the "symptom-based strategy" of the CDC [25].

Regarding detection of the viral nucleic acid by RT-qPCR in NPS and induced sputum samples on the third day of symptoms, 86.7% of cases had positive NPS, and 80.0% had positive induced sputum samples. A few patients (10.0%) had positive NPS and negative induced sputum samples. The positive rate of sputum samples on the 3rd day was lower than that of NPS, with a highly significant statistical difference (*P* = 0.001).

This finding mismatched with Lin et al. [26] who found that the positive rate of sputum specimens was higher than that of nose and throat swabs (76.9% and 44.2% respectively).

This may be explained by the slightly high percentage of mild cases (58.3%) in our study who presented most frequently with upper respiratory symptoms of COVID-19 infection rather than lower respiratory ones. Although sputum is one of the lower respiratory tract samples, it gives a better positive result in a patient with lower respiratory tract infections. Also, in the Lin et al. [26] study, the average age of the study population was 57.3 years. As a result, elderly patients presented with more severe forms of the disease, including lower respiratory tract infections, while the median age of our study population was 32 years old.

While the results of viral nucleic acid by RT-qPCR in NPS and induced sputum samples on the 10th day of symptoms showed a higher positive rate in induced sputum samples than NPS [19 (31.7%), 14 (23.3%)], only three (5.0%) of the 19 positive induced sputum sample cases were positive by NPS, while the remaining 16 (26.7%) had positive induced sputum and negative NPS results. This is most likely owing to the greater prevalence of angiotensin converting enzyme-2 (ACE2) receptors in pneumocytes and epithelial cells of the lower respiratory airway relative to upper airway epithelial cells. That had been recognized as the SARS-CoV-2 functional receptor [27, 28]. This finding agrees with Liu et al. [29] who found longer SARS-CoV-2 detection times in sputum samples compared to NPS samples. According to Zhang et al. [30] the findings were also similar.

Early in the disease, results of high viremia were found more in NPS than in induced sputum samples (41.7% vs. 38.3%, respectively). In contrast, at day 10, more cases with high viral loads were seen in induced sputum than in NPS (15.0% vs. 10.0%, respectively). The length of time between virus shedding may vary from part of the respiratory tract to another [30, 31]. For example, in the upper respiratory tract specimens, peak levels of SARS-CoV-2 were seen very early in the course of the disease [31]. Moreover, another study reported that viral shedding occurs over a longer period of time in lower respiratory tract secretions and that the maximum viral loads appear about 2 weeks after the onset of symptoms [32]. In these regions, the different expression levels of ACE2, the putative cell entry receptor of SARS-CoV-2, may partly explain this phenomenon [27, 28]. Meanwhile, the lower respiratory tract communicates less with the outside world than the upper respiratory tract, exacerbating viral retention [33, 34].

Regarding the median CT value on the 3rd day, we found an insignificant difference between NPS results and induced sputum results (*p* value = 0.308). Our result showed agreement with Liu et al. [29] who compared SARS-CoV-2 CT values in sputum and NPS samples with and without underlying diseases, and the p values were 0.65 and 0.22, respectively [29].

By using the ROC curve, we found that the overall diagnostic performance of induced sputum was relatively greater than NPS with a statistically significant difference (*p* value = 0.0418), The sensitivity, specificity, PPV, and NPV of induced sputum were 64.0%, 61.43%, 54.2%, and 70.5%, respectively, compared to NPS (58.0%, 55.71%, 48.3%, and 65.0%, respectively). This was matched with the findings of Lai et al. [35], who discovered that induced sputum samples outperformed oropharyngeal swabs in terms of sensitivity and specificity (85.5% and 79.1%, respectively). There was a statistically significant positive correlation between viral load according to the CT values of both samples at day 3 (r = 0.497, *p* = 0.000).

## Conclusion

Induced sputum and NP swab tests had comparable results. In terms of diagnosing COVID-19, NPS was slightly superior to sputum early in the disease course, yielding more positive results. Later in the disease course, an induced sputum sample yielded higher sample positivity and viral load than NPS. Furthermore, induced sputum collection is a straightforward, non-invasive, and risk-free method.

We believe that sputum might aid in identifying COVID-19 in sputum-producing patients. Therefore, induced sputum could be useful for COVID-19 confirmation in patients with radiologically or epidemiologically suspected COVID-19 who have a negative NPS or in difficult-to-diagnose COVID-19 patients. Negative induced sputum should be used as a criterion for hospital discharge for COVID-19 recovering patients and release from quarantine. However, this study does not fully invalidate the need for nasopharyngeal sampling but rather recommends a noninvasive, relatively simple collection method with apparent advantages, such as induced sputum samples.

The small sample size, single-center study, and restriction of sample types to NPS and induced sputum samples were the study's limitations, as other samples would have been better tested. As an example, saliva is simple, quick, and safe to collect. Moreover, new research has indicated that saliva has some advantages in COVID-19 diagnosis [14, 15, 36].

## Data Availability

All data generated or analyzed as part of this study are included in this published article.

## Abbreviations

CDC: Centers for Disease Control and Prevention
COVID-19: Novel coronovirus disease for 2019
CRP: C‐reactive protein
CT: Cycle threshold
HB: Hemoglobin
NPS: Nasopharyngeal swabs
ROC: The receiver operating characteristic
RT-qPCR: Real-time quantitative reverse transcriptase-polymerase chain reaction.
SARS-CoV-2: Severe acute respiratory syndrome coronavirus 2
TLC: Total leucocytic count
WHO: The World Health Organization
